# Survey on experiences and attitudes of parents toward disclosing information to children with genetic syndromes and their siblings in Japan

**DOI:** 10.1038/s41598-022-19447-3

**Published:** 2022-09-08

**Authors:** Mikiko Kaneko, Daiju Oba, Hirofumi Ohashi

**Affiliations:** Division of Medical Genetics, Saitama Children’s Medical Center, 1-2, Chuo-ku Shintoshin, Saitama-shi, Saitama 330-8777 Japan

**Keywords:** Genetics, Psychology, Diseases

## Abstract

Many parents face the dilemma of when, how, and what to disclose to their children regarding their genetic conditions. The purpose of this study was to learn about the experiences of parents regarding disclosing information to their children with genetic conditions. A questionnaire was sent to 378 parents of children and adolescents with the following genetic syndromes: 22q11.2 deletion syndrome, Beckwith–Wiedemann syndrome, Noonan syndrome, Russell–Silver syndrome, Kabuki syndrome, Williams syndrome, Prader–Willi syndrome, and Sotos syndrome. Findings were analyzed using descriptive statistics for multiple-choice questions. Of the parents surveyed, 158 (41.8%) responded to the questionnaires. The average age of children with genetic syndromes was 12 years. Sixty-seven parents had disclosed relevant information to their children, whereas 91 had not. Among them (who had disclosed information), out of 53 respondents who answered that their affected child had siblings, 50 had disclosed the genetic condition of the affected child to the siblings as well. Sixty-eight out of 91 respondents who had not told information to affected child were planning to disclose the information in the future. Many respondents who had disclosed information did not regret this. They felt good talking about genetic conditions, and had talked about genetic conditions with the affected children following disclosure. This study contributed to our understanding of the attitudes of parents towards disclosing information to children with genetic syndromes.

## Introduction

Following the diagnosis of a child with a genetic condition, parents face the challenge of disclosing the nature of the condition with their affected child. The disclosure includes diagnosis, symptoms and management such as hospitalization and commuting to the hospital. Many parents face the dilemma of when, how, and what to disclose to their children about their genetic conditions^1–3^. Most parents express emotions such as anxiety, worry, and concern, with many relying on their own experiences regarding a genetic condition in the family for informing their own children^4^. Parents who did not share any information tended to evaluate their children as being too young or immature to understand the information^2^. However, disclosing information to children about their genetic condition is important if the affected children are to lead autonomous and independent lives in the future.

It has been shown that children who gradually learned about their conditions as they grew up could more efficiently cope and come to terms with the risks of either to themselves or to other family members^5^. Parents and adolescents said that receiving information about their genetic conditions as younger children was less shocking, and even more so when they were gradually informed of all associated aspects as they grew up^6^. Therefore, disclosing such information from an early age is important and helps children understand the nature of their health condition and its more efficient management.

However, the rates of disclosure to children about their genetic conditions might not be high. In a previous study in United States, 68 out of 139 participants (49%) reported sharing information about the condition and its inheritance and 57 out of 139 participants (41%) reported selectively sharing information with affected children^2^.

As such mentioned above, most studies concerning disclosing information to children with genetic conditions were from United States or European countries. Such studies are scarce in Japan. Therefore, we conducted a cross-sectional study by using a questionnaire survey to investigate the experiences and attitudes of parents regarding the disclosure of medical information to children and adolescents with genetic conditions and learn the ways through which such information should be disclosed to children and their siblings. In this cross-sectional study, we planned to obtain data for multiple-choice questions, such as whether or not disclosed information, if disclosed, when, who, how, reasons and details for disclosure, if not disclosed, the reasons for avoiding disclosure, whether or not they will disclose information.

## Methods

### Editorial policies and ethical considerations

This study was approved by the Saitama Children’s Medical Center Institutional Review Board (#2019-02-020) and all methods were performed in accordance with the relevant guidelines and regulations. Questionnaires were provided along with an informed consent form. Upon consent, the parents participated in our study by returning the completed questionnaires together with the signed consent form.

### Design

We conducted a cross-sectional study by using a questionnaire survey between November and December 2019. The questionnaire was multiple-choice questions about the disclosure of information to children and adolescents with genetic conditions.

### Participants

A number of group programs for genetic syndromes have been conducted at the outpatient clinic of Saitama Children’s Medical Center (SCMC) since 2000. Potential participants were identified through a list of families from these programs. Questionnaires were sent to 378 parents or caregivers of children with one of the following genetic syndromes: 22q11.2 deletion syndrome (22qDS), Beckwith–Wiedemann syndrome (BWS), Noonan syndrome (NS), Russell–Silver syndrome (RSS), Kabuki syndrome (KS), Williams syndrome (WS), Prader–Willi syndrome (PWS), or Sotos syndrome (SS). These eight genetic syndromes were chosen among those for which group clinics have been regularly held in our hospital in regard to their generally less severe or no intellectual disability, considering appropriateness for this survey (brief description of each condition is shown in Supplementary Table S1).

We excluded children who were less than 4 years old or who were diagnosed within 1 year of enrollment from the study.

### Measures

A common questionnaire was prepared, by which all parents were asked whether they had disclosed information to their children (Table 1, see Supplementary Table S2 in detail). Precise definition of disclosing such as regarding diagnosis, symptoms, cause of genetic condition, management, heredity and reason for going to the hospital was not provided here, but instead we asked participants whether they have told their children about any information of their genetic conditions. If the answer was “Yes”, the parents moved to Questionnaire I and were asked more in detail (Table 1). If the answer was “No”, the parents moved to Questionnaire II for further questions (Table 1).

**Table 1:** Questionnaires.

**Common questionnaire for all respondents**	
Q1	Name, relationship, and age of respondent
Q2	Name, sex, birth order, age, diagnosis, and age of diagnosis of child
Q3	Whether parents disclosed information or not If yes, move to questionnaire I If no, move to questionnaire II
**Questionnaire I. For respondents who had disclosed information to their affected children**	
Q1	The age of the child at disclosure
Q2	Who primarily carried out the disclosure?
Q3	Who was present other than the above persons?
Q4	Reasons for disclosure
Q5	Details and important points when disclosing
Q6	Whether or not the genetic condition was discussed after disclosure
Q7	Information that was helpful for disclosure
Q8	How did you feel after the disclosure?^a^
Q9	Number of siblings of the child with a genetic condition
Q10	Whether parents disclosed information about genetic conditions to the siblings or not
Q11	The age of siblings at disclosure, who primarily carried out the disclosure, who was present, reasons, details, and important points when disclosing
Q12	Whether or not the genetic condition was discussed with the siblings after disclosure
Q13	Will you disclose to the siblings later, if you haven’t yet?
Q14	When will you disclose to the siblings?
Q15	Your plan to disclose to the siblings: age at disclosure, who will primarily carry out the disclosure, who will be present, reasons, details, and important points when disclosing
**Questionnaire II. For responders who had not disclosed information to affected children**	
Q1	Reasons for avoiding disclosure
Q2	Whether or not you will disclose information in the future
Q3	If you plan on disclosing in the future: the age at disclosure, who will primarily carry out the disclosure, who will be present, reasons, details, and important points when disclosing
Q4	Number of siblings of the child with a genetic condition
Q5	Whether parents disclosed information about genetic conditions to siblings or not
Q6	The age of siblings at disclosure, who primarily carried out the disclosure, who was present, reasons, details, and important points when disclosing, OR your plan to disclose information to the siblings

^a^Respondents were asked to indicate the degree of their feelings by choosing any number on a scale of 0–100.

Questionnaire I (for respondents who disclosed information to their children) contains 15 questions, and Questionnaire II (for respondents who had not disclosed information to their children) contains 6 questions, respectively (Table 1). All questions are multiple-choice questions with spaces where respondents can explain in detail. On <Q8> of Questionnaire I, respondents were asked to indicate the degree of their feelings by choosing any number on a scale of 0–100, with the lower and upper extremes.

### Data analysis

We used descriptive statistics to analyze multiple-choice questions which we report on this study.

## Results

### Common questionnaire for all respondents

Of the 378 parents, 158 responded to the survey (response rate: 41.8%). The respondents consisted of 128 mothers, 11 fathers, and 19 both parents. The average age of respondents was 45 years. The current age of children and their age at the time of diagnosis of their genetic condition were 12 years and 2 years old on average, respectively. Information on respondents and their children are shown in Table 2.

**Table 2:** Classification of responders, sex, diagnosis, and age of children, and number of siblings.

	%	n	Average age at the participation in this study (range)	Age at diagnosis (range)
**Responders (N = 158)**			45 (25–65)	
Father	7	11		
Mother	81	128		
Parents	12	19		
**Diagnosis**^**a**^				
22q11.2 deletion syndrome		27	11 (4–28)	2 (0–8)
Beckwith–Wiedemann syndrome		19	14 (4–28)	< 1 (0–1)
Noonan syndrome		17	11 (4–28)	4 (0–12)
Russell–Silver syndrome		3	5 (4–7)	1 (1–5)
Kabuki syndrome		29	14 (4–28)	2 (0–11)
Williams syndrome		28	13 (4–26)	2 (0–11)
Prader–Willi syndrome		19	12 (4–23)	1 (0–10)
Sotos syndrome		16	11 (4–24)	1 (0–8)
Total		158	12 (4–28)	2 (0–11)
**Sex of child**				
Male	58	91		
Female	42	67		
**Disclosure**				
Disclosed	42	67		
Not disclosed	58	91		
**Siblings**^**b**^				
One or more siblings	81	128		
One sibling		91		
Two siblings		34		
Three siblings		3		
No siblings	19	30		

^a^The diagnosis and ages of each child with a genetic condition.

^b^Ages of siblings neither at the participation in the study nor at the diagnosis of children with genetic condition was not provided. Ages at disclosure of genetic condition are shown in Fig. 2.

**Figure 1:**
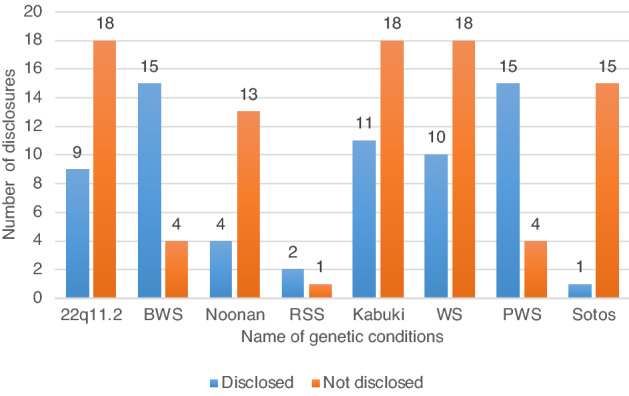
Number of disclosures by genetic condition. Note this number reflects children with genetic conditions.

Of the 158 respondents, 67 (42%) had disclosed information to their children regarding their genetic conditions, whereas 91 (58%) did not. Children with BWS (79%), PWS (79%) and RSS (67%) were more likely informed, whereas children with KS (38%), WS (36%), 22DS (33%), NS (24%), and SS (6%) were less likely informed as shown in Fig. 1.

### Questionnaire I

#### Respondents who had disclosed information for affected children

**Figure 2:**
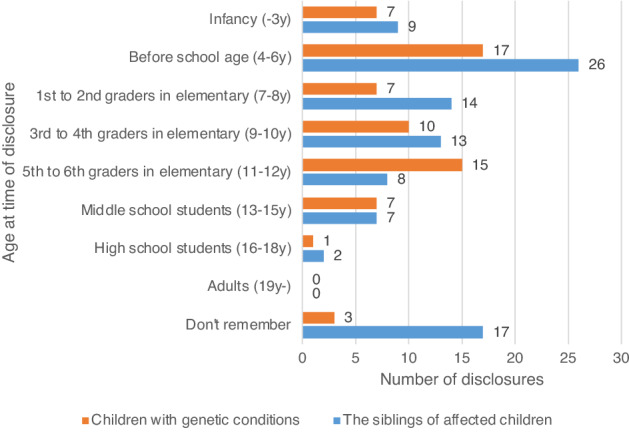
Age at time of disclosure. Note the number of the siblings of affected children reflects the total number of respondents who answered that they had disclosed information to the siblings of affected children both in questionnaire I (for respondents who had disclosed information to affected children) and in questionnaire II (for those who had not).

The most frequent answer regarding the time at which respondents talked about genetic conditions for the first time was when their affected children were at preschool age (4–6 years). The second most frequent response was when children were around the fifth to sixth grades in elementary school (11–12 years). No one disclosed the information after the children had reached 19 years of age (Fig. 2).

The mother was the primary person disclosing the diagnosis (n = 35; 52%). Disclosure by both parents was the second most frequent answer (n = 25; 37%), whereas disclosure by the father constituted only 3% (n = 2) of answers. Opportunities of disclosure other than parents, such as doctors, group meetings, and illustrated books, constituted 10% (n = 7) of answers.

The most common reason behind their decision to reveal the diagnosis to their children was that respondents wanted their affected children to understand the reason for their more frequent visits to the hospital compared with their peers (n = 29; 43%) (Table 3). The next most frequent reason for that was that children were mature enough to understand (n = 20; 30%). Other answers that respondents freely described included the following: "I want him/her to know and understand him/herself", "he/she will have to have strength to face issues concerning his/her condition in the future", "we had to convince him/her to have his/her surgery", "his/her friends started to ask him/her naturally about physical features that other peers do not have and he/she should be able to answer on his/her own", "he/she already had some knowledge of it from group meetings, so it was time anyways".

**Table 3:** Reasons for disclosure.

Items		Children with genetic condition (n = 67)		Siblings^a^ (n = 50)	
		%	n	%	n
1	Asked about the symptoms	13	9	16	8
2	Asked why he/she visited the hospital	25	17	30	15
3	Thought he/she was old enough to understand	30	20	44	22
4	Wanted him/her to understand why he/she visited the hospital as a result of disclosing information	43	29	36	18
5	Seemed to be concerned about physical symptoms	7	5	10	5
6	Because his/her friends said something	10	7	14	7
7	Because of an event, such as enrollment, employment, or marriage	16	11	12	6
8	Other (if possible, please explain in detail)	37	25	26	13

Note all items were multiple-choice answers.

^a^This number reflects the number of respondents who answered that they had disclosed information to the siblings of affected children in questionnaire I.

The most common detail which was discussed with their children at the time of disclosing the information of their diseases was symptoms (n = 49; 73%), with diagnosis being the next most common (n = 42; 63%) (Table 4). Other details freely described included "the condition is no one’s fault" and "everyone, not only you, has strengths and weaknesses".

**Table 4:** Details of disclosed information.

Items		Children with genetic condition (n = 67)		Siblings^a^ (n = 50)	
		%	n	%	n
1	Diagnosis	63	42	76	38
2	Symptoms	73	49	92	46
3	Cause of genetic condition	16	11	28	14
4	Management	49	33	50	25
5	Heredity	7	5	36	18
6	Reason for going to the hospital	57	38	54	27
7	Other (if possible, please explain in detail)	9	6	2	1

Note all items were multiple-choice answers.

^a^This number reflects the number of respondents who answered that they had disclosed information to the siblings of affected children in questionnaire I.

The most frequently chosen important point when disclosing the disease information to their children was their attempt to explain in a way that was easy to understand (n = 45; 67%) (Table 5). Keeping descriptions simple (n = 33; 49%) and trying to provide children with a sense of relief (n = 32; 48%) were the next most important points. Other points freely described included “telling children that they were not alone”, “telling them that this was something to face together with the whole family”, “telling them that they would be helped by a lot of people, encouraging them to be positive and regard their condition as an aspect of their personality and individuality”, “telling them that there were various disorders”, and “making it clear that everyone has their own challenges in life”.

**Table 5:** Important points when disclosing.

Items		Children with genetic condition (n = 67)		Siblings^a^ (n = 50)	
		%	n	%	n
1	Be honest	36	24	56	28
2	Keep it simple	49	33	44	22
3	Try to explain in a way that is easy to understand	67	45	66	33
4	Be direct; use appropriate terms	3	2	6	3
5	Do not treat it as an abnormality	27	18	36	18
6	Try to be relieved	48	32	38	19
7	Treat it as "no big deal"	25	17	24	12
8	Other (if possible, please explain in detail)	15	10	8	4

Note all items were multiple choice answers.

^a^This number reflects the number of respondents who answered that they had disclosed information to the siblings of affected children in questionnaire I.

In terms of whether respondents continued discussing with their children about the genetic condition after disclosing the diagnosis, 12% (n = 8) of respondents chose "never", whereas 85% of them chose "sometimes" (n = 29; 43%) or "always" (n = 28; 42%). The reasons for "never" were that respondents had just told the child, the child did not understand yet, the child did not talk about it, or the child did not ask about it. In the case of "sometimes", respondents had talked about the condition on occasions such as hospital visits, hospitalization, or group meetings, whenever the child asked about it, whenever the child experienced trouble regarding the condition, or when it had become a topic of conversation. The reasons for "always" were that respondents talked whenever the child was in trouble, on occasions such as participating in group studies, when watching TV, whenever the child asked about, whenever the child seemed to be concerned about symptoms, for the sake of the child’s future, or because they had to provide the child with solutions for dealing with troubles regarding their conditions.

Respondents had received useful information on disclosing from attending doctors (n = 41; 61%), from patients and family associates (n = 32; 48%), from the internet (n = 16; 24%), and from other sources (n = 10; 15%). Other sources they considered useful included such as documents received from attending doctors about treatment or surgery, and related books.

**Figure 3:**
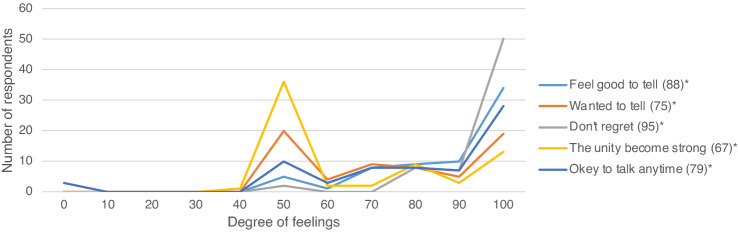
Degree of feelings of respondents after disclosing information. Note respondents chose any number on a scale between 0 (lowest) and 100 (highest) as indicating the degree of their feelings. *Mean values for each items.

The degree of their feelings after disclosure were the following: the lowest reported, highest reported, and mean values for each item were 50, 100, and 88 for item 1(“feel good to tell”), 50, 100, and 75 for item 2 (“wanted to tell”), 50, 100, and 95 for item 3 (“don’t regret”), 40, 100, and 67 for item 4 (“the unity become strong”), and 0, 100, and 79 for item 5 (“okey to talk anytime”), respectively. Many respondents felt disclosing information about the condition was a good thing and did not regret doing so (Fig. 3). In contrast, a few respondents felt difficulty talking anymore with their families about the genetic condition. Other feelings and thoughts freely reported by respondents included; “Better to know about the child’s genetic condition oneself.”, “Wish the child would think of it as one of his/her personalities.”, “By knowing and understanding one’s genetic condition, the child would ask for help when he/she needs it and be able to deal with problems.”, “Nothing to be ashamed of.”, and “Thought the child was old enough to understand and the timing was right to tell” .

#### Respondents who also had disclosed information for siblings

Of the 53 respondents who answered that their affected child had siblings, 50 had disclosed the genetic condition of the affected child to the siblings.

Regarding the time of disclosure, preschool age (4–6 years) was also the most frequent response for siblings. However, inability to remember when they first informed the siblings was the second most frequent response. Mothers were the ones that primarily disclosed the relevant information (n = 35; 70%). Disclosure by both parents was the next most frequent answer (n = 12; 24%), followed by disclosure by other resources, such as doctors, grandmothers, or group meetings (n = 5; 10%) and fathers (n = 3; 6%).

The most common reason (Table 3) for their decision to inform siblings was that respondents thought they were mature enough to understand (n = 22; 44%). The second most frequent answer was that they wanted the sibling to understand the reason for the frequent visits of the affected children to the hospital (n = 18; 36%). Other reasons included the following: "I wanted the sibling to understand about the genetic condition and needed his/hers cooperation", "I was dissatisfied with how differently I dealt with the child with the genetic conditions", "the sibling ridiculed the physical characteristics of the child with the genetic conditions", "the sibling started to notice the difference between the siblings of his/her friends and his/her own sibling", "I just naturally found myself wanting to talk".

The most common detail (Table 4) of the disclosure of such information was symptoms (n = 46; 92%) and the next most common was the diagnosis (n = 38; 76%) of the affected child.

The most frequently chosen important point (Table 5) when disclosing this information was that respondents tried to explain in a way that was easy to understand (n = 33; 66%). Being honest was the second most frequently chosen important point (n = 28; 56%). Other important points reported by respondents included: "I praised the sibling for also working hard during the surgery and hospitalization of the child with the genetic condition", "we told the sibling that if he/she would cooperate, the child with the genetic condition could become independent", "I told the sibling that the child with the genetic condition had an intellectual disability", and "children with genetic conditions might sometimes be restricted from engaging in things they want to do, but there should also be a bright future for them too".

About whether respondents continued discussing on the genetic condition after disclosure, 10% (n = 5) of respondents (total number of respondents was 49, excluding one who did not answer this question) chose "never", whereas 90% of them chose "sometimes" (n = 23; 47%) or "always" (n = 21; 43%). The reasons for choosing "never" included not asking about it, not seeming to care, or because parents did not view it as something special, so there was no need to talk about it anymore.

### Questionnaire II

#### Respondents who had not disclosed the diagnosis to affected children yet

The average age of children with a genetic condition who had not been told about his/her condition was 10 years old at the participation in this study.

The most common reason for not disclosing the diagnosis was that the children were not mature enough to understand (n = 70; 77%). The next most frequent answer was "other" (n = 28; 31%), followed by "had never been asked about symptoms" (n = 23; 25%). As it concerns the reasons given for "other", 22 (79%) out of 28 respondents mentioned difficulty with understanding because of intellectual disability. One responder was afraid that if they disclosed the disorder, the child would unintentionally expose its condition to others, which was not desired by the parents. Another responder answered that they did not feel the need to disclose the diagnosis at this point.

Regarding whether they would disclose information to their children in the future, 68 (75%) out of 91 respondents answered that they plan to disclose the diagnosis in the future, 24% (n = 22) answered that they would not, and 1% (n = 1) answered that they were not sure. The reasons for disclosing information in the future were described as such, including "he/she can live a better life if he/she knows, " "he/she will be able to get appropriate support if he/she knows the characteristics of the disorder", "we want him/her to live life with a positive outlook by understanding and accepting the disorder", "we want him/her to be able to communicate his/her needs and ask for help from others when needed", "we will tell him/her why he/she is different from his/her peers when he/she notices and asks about that", "we will tell him/her what he/she should know when he/she starts asking because it’s not a matter that should be hidden", "it will be necessary when getting married and starting a family".

Reasons for not disclosing the information in the future included the difficulty of the child to understand because of intellectual disability, the unknown time by which the child would be able to understand, or the belief of respondents that it was not a matter to deal with proactively. One responder answered, "I am not sure whether to disclose the information even in the future because nobody knows when they will have grown enough to understand their diagnosis. If they could understand, I would want to tell them, but no one knows when that will be". Regarding the age at which they would disclose this information to the affected child in the future, 32 out of 68 respondents (47%) planning to tell the child about their condition answered "uncertain"; which was the most frequent answer. The second most frequent answer was "third to fourth grader in elementary school (9–10 years)". No one mentioned "19 years old or older".

The most frequent answer regarding who will primarily carry out the disclosure of the information to affected children was both parents (n = 43; 63%), with the mother being the second most frequent answer (n = 22; 32%), whereas the father constituted only 7% (n = 5), and others, such as attending doctors and grandparents constituted 4% (n = 3) and 1% (n = 1) of all answers, respectively.

Regarding siblings, among 75 respondents who had siblings, 34 (45%) had already informed them. Out of remaining 41 respondents who had not informed, 35 (47%) respondents planned to disclose the information in the future, four (5%) respondents would not disclose the information, and two (3%) declined to answer.

## Discussion

We conducted a survey on parents/caregivers of children with eight genetic syndromes, consisting of 22qDS, BWS, NS, RSS, KS, WS, PWS, and SS, regarding the disclosure of information about their conditions. Of 378 parents surveyed, 158 (41.8%) responded to the questionnaires. Although the rate of disclosure was rather low (less than half; n = 67, 42%), many respondents who had disclosed information had not regretted this and felt it was an ultimately good decision.

The rate of disclosure in our study cohort (42%) seems similar to previous findings. For instance, as mentioned earlier, Gallot et al., reported that, of the 139 parents, almost half (n = 68; 49%) openly shared information about the genetic condition with their children^2^. In our study, respondents whose children had a genetic condition, such as BWS or PWS, had higher rates of disclosing information compared with those of children with other genetic conditions. We assumed that the level of intellectual disability of the child might affect whether this information would be disclosed to them. Children with BWS have no intellectual disability, and visit the hospital regularly (e.g., abdominal ultrasound) even in the absence of symptoms; therefore, an explanation for the reason of these visits might be highly required. Children with PWS require a strong effort or cooperation from the individual in terms of dietary management, which likely influences the high necessity for explanation. However, we were unable to identify the reasons behind the differences in the rate of disclosure for these genetic conditions.

In our study, respondents most frequently disclosed the diagnosis in early childhood during preschool ages (4–6 years old). Szybowska^7^ reported that adolescents prefer to receive information about the genetics of their condition at a much younger age (6–10 years old) than when they are usually seen for genetic counseling. Being informed at an early stage of childhood might help them understand the nature of their health condition and its more efficient management.

Many respondents in our study talked about diagnoses, symptoms, and management of their children, but rarely about the cause of a genetic condition or its inheritance, which seemed to concur with the notion reported by Gallo^8^, who pointed out that parents do not want to address the potential negative consequences of the condition or its inheritance.

Respondents in our study tried to explain in a way that was easy to understand, honest, and positive. They also tried to dispel worries and anxiety as much as possible. This is in line with previous studies. Dennis^9^ recommended using simple and direct language and avoiding ambiguous descriptions or complex terminology, and that parents be honest with the child and stay positive as well. In a study about informing girls with Turner syndrome regarding their medical condition, Takahashi^10^ reported that most parents seemed to share information with their child in a positive manner.

Many respondents (85%) in our study continued to discuss the genetic condition of the affected children following disclosure, while some respondents (12%) answered that they “never” discussed about it. Metcalf et al.^4^ reported similar findings that parents who openly communicated with their children never expressed regret about discussing the genetic condition with their families, while parents sometimes reported feeling afraid to discuss their child’s emotions of worry, depression, frustration, or embarrassment. However, the reasons of our participants for not talking with their children after disclosure was not because of worry or fear, instead simply because that there existed no chance or necessity to talk about it. Therefore, vast majority of our respondents who disclosed disease information to their children were able to maintain sharing information well among their families without adverse consequences. Further research is needed to assess whether this attitude is characteristic unique to Japanese families.

More than half of respondents who had disclosed the diagnosis to their affected children thought that advice from the attending doctor on what and how to disclose the information was helpful. They also received useful information from the associations of patients and families in our study. Dana et al.^11^ reported that many parents who had disclosed the diagnosis to their child with 22qDS felt they could have benefited from additional advice from professionals to increase their confidence and success of the disclosure. Societal organizations, such as associations of patients and families can play a very important role in helping to solve the problems of parents by supporting each other^10^.

We also found that many respondents (n = 68, 75%) who had not yet disclosed information to their children planned to do so in the future when the children grew to be able to understand what they should know. The most common reason for not disclosing was the intellectual disability of the child, which was fully understandable. On the other hand, Dana et al.^11^ reported that parents who had not informed children with 22qDS were uncertain about the words to use, how to initiate the conversation, or were concerned about the level of understanding of their child. It may be important for healthcare providers to talk with parents in advance regarding such as timing, attitude, and understandability of the message to be conveyed on disclosing information to their children in the future.

This study provides the actual experiences and attitudes of parents regarding disclosing information to children with genetic conditions and their siblings in Japan. We think that one of the most important messages of this survey is the fact that vast majority of respondents felt good to tell information about the condition and had no regret. We hope that information of this findings would help parents of children with genetic conditions when they are thinking about disclosing genetic conditions to children.

There were several limitations to this study. The time from diagnosis varied, and the ages of children ranged from 4 to 28 years. Whether parents had disclosed information to their affected children and siblings could vary depending on the time from diagnosis and age of children. All children included in the study had a genetic syndromes commonly associated with intellectual disability. However, the degree of intellectual disability varies and some had only subtle or no intellectual disability. The extent of a child’s intellectual disability may also affect the disclosure of information to them. It is difficult to determine the extent to which this study can be generalized to a wider population of families affected by these and other genetic conditions. Additionally, this study did not explore the perspectives of children with genetic condition or their siblings. Further study on disclosing information about the diagnoses of children with a focus on the children themselves is necessary for parents who are planning to disclose information to their children.

## Supplementary Information

Supplementary Table S1.

Supplementary Table S2.

## Data Availability

All data generated or analyzed during this study are included in this published article and its supplementary information file.
