# Supplementary material for: Survey on experiences and attitudes of parents toward disclosing information to children with genetic syndromes and their siblings in Japan
Source: Sci Rep. 2022 Sep 8;12:15234. doi: 10.1038/s41598-022-19447-3 (PMC9458639; doi:10.1038/s41598-022-19447-3)
Supplement: Supplementary file 1 — Supplementary Table S1. [file 41598_2022_19447_MOESM1_ESM.pdf]

Survey on experiences and attitudes of parents toward disclosing information to children with genetic syndromes and their siblings in Japan

Mikiko Kaneko[1], Daiju Oba[1], Hirofumi Ohashi[1]*

[1] Division of Medical Genetics, Saitama Children's Medical Center, Saitama, Japan

*Correspondence
Hirofumi Ohashi, MD, Ph.D
Division of Medical Genetics, Saitama Children's Medical Center
1-2, Chuo-ku Shintoshin, Saitama-shi, Saitama 330-8777, Japan
Tel.: +81-48-601-2200
Email: ohashih@peach.ocn.ne.jp

Supplementary Table S1. Description of each genetic syndrome

22qDS	A chromosomal microdeletion syndrome characterized by growth and developmental delay, cardiac defects, palatal anomalies, psychiatric disorders, and rarely immunodeficiency.
BWS	A condition characterized by overgrowth, macroglossia, hemihyperplasia and tumor predisposition. Intelligence is not impaired in the condition.
NS	A multisystemic disorder typically characterized by short stature, congenital heart defects, cardiomyopathy, and skeletal abnormalities. Most individuals with NS have normal intelligence, but some have mild to moderate intellectual disability.
RSS	A condition characterized by prenatal and postnatal growth failure, skeletal asymmetry and short incurved fifth fingers. Delayed motor and speech development are also a feature.
KS	A condition characterized by typical facial features, mild to moderate intellectual disability and multisytemic defects such as congenital cardiac defects and cleft lip/palate.
WS	A chromosomal microdeletion syndrome characterized by intellectual disability, cardiovascular diseases (typically supravalvular aortic stenosis), endocrine abnormalities and spinal curvature abnormalities. Weakness in visual-spatial processing, hypersensitivity to sounds, affinity for music and friendly social personality are also characteristics.
PWS	A condition characterized by neonatal feeding difficulties due to severe hypotonia and later obesity with hyperphagia. Individuals with PWS typically have mild to moderate intellectual impairment and behavioral problems, including temper outbursts, stubbornness, and compulsive behavior.
SS	A condition characterized by overgrowth in infancy, macrocephaly and systemic abnormalities such as scoliosis. Most individuals with SS have intellectual disability and behavioral disturbance such as phobias, obsessions and compulsions, tantrums, and impulsiveness.

22qDS 22q11.2 deletion syndrome, *BWS* Beckwith-Wiedemann syndrome, *NS* Noonan syndrome, *RSS* Russell-Silver syndrome, *KS* Kabuki syndrome, *WS* Williams syndrome, *PWS* Prader-Willi syndrome, *SS* Sotos syndrome,
