# Supplementary material for: Survey on experiences and attitudes of parents toward disclosing information to children with genetic syndromes and their siblings in Japan
Source: Sci Rep. 2022 Sep 8;12:15234. doi: 10.1038/s41598-022-19447-3 (PMC9458639; doi:10.1038/s41598-022-19447-3)
Supplement: Supplementary file 2 — Supplementary Table S2. [file 41598_2022_19447_MOESM2_ESM.pdf]

Survey on experiences and attitudes of parents toward disclosing information to children with genetic syndromes and their siblings in Japan

Mikiko Kaneko[1], Daiju Oba[1], Hirofumi Ohashi[1]*

[1] Division of Medical Genetics, Saitama Children's Medical Center, Saitama, Japan

*Correspondence
Hirofumi Ohashi, MD, Ph.D
Division of Medical Genetics, Saitama Children's Medical Center
1-2, Chuo-ku Shintoshin, Saitama-shi, Saitama 330-8777, Japan
Tel.: +81-48-601-2200
Email: ohashih@peach.ocn.ne.jp

Supplementary Table S2. Questionnaires

Common questionnaire for all respondents	
Q1	Name, relationship, and age of respondent
Q2	Name, sex, birth order, age, diagnosis, and age of diagnosis of child
Q3	Whether parents disclosed information or not<br>If yes, move to questionnaire I<br>If no, move to questionnaire II
Questionnaire I. For respondents who had disclosed information to their children	
Q1	The age of the child at disclosure
Q2	Who primarily carried out the disclosure?<br>Items:<br>1. Father 2. Mother 3. Parents<br>4. Ground father 5. Ground mother 6. Ground parents<br>7. Health care providers (if possible, please explain in detail)<br>8. Other (if possible, please explain in detail)
Q3	Who was present other than the above persons?<br>Items:<br>1. Father 2. Mother<br>3. Ground father 4. Ground mother<br>5. Siblings (older brother, older sister, younger brother, younger sister)<br>6. Other (if possible, please explain in detail)
Q4	Reasons for disclosure<br>Items:<br>1. Asked about the symptoms<br>2. Asked why he/she visited the hospital<br>3. Thought he/she was old enough to understand<br>4. Wanted him/her to understand why he/she visited the hospital as a result of disclosing information<br>5. Seemed to be concerned about physical symptoms<br>6. Because his/her friends said something<br>7. Because of an event, such as enrollment, employment, or marriage.<br>8. Other (if possible, please explain in detail)
Q5	Details and important points when disclosing

Items for details of disclosure:

1. Diagnosis
2. Symptoms
3. Cause of genetic condition
4. Management
5. Heredity
6. Commuting to the hospital

Items for important points:

1. Be honest
2. Keep it simple
3. Try to explain in a way that's easy to understand
4. Be direct; use appropriate terms
5. Do not treat it as an abnormality
6. Try to be relieved
7. Treat it as "no big deal"
8. Other (if possible, please explain in detail)

Q6 Whether or not the genetic condition was discussed after disclosure

Items:

1. Always (if possible, please explain reasons)
2. Sometimes (if possible, please explain reasons)
3. Never (if possible, please explain reasons)

Q7 Information that was helpful for disclosure

Items:

1. From attending doctors
2. From patients and family associates
3. From the internet
4. From other sources

Q8 How did you feel after the disclosure? [1]

Items:

1. a scale of 0 "the outcome of disclosure was not good for us at all" to a scale of 100 "the outcome of disclosure was extremely good for us"
2. a scale of 0 "I really hesitated to tell them their diagnosis" to a scale of 100 "I really wanted to tell them the whole story for a long time"
3. a scale of 0 "I really regret telling them the diagnosis" to a scale of 100 "I will never regret telling them the diagnosis"
4. a scale of 0 "my family is not functioning well" to a scale of 100 "family ties are stronger than ever before"
5. a scale of 0 "it's not easy to talk about it anymore" to a scale of 100 "we can talk about it anytime we want to"
6. Other (Please share any specific feelings or thoughts you have regarding disclosure.)

Q9 Number of siblings of the child with a genetic condition

Q10 Whether parents disclosed information about genetic conditions to the siblings or not

Items:

1. Yes 2. No

Q11 The age of siblings at disclosure, who primarily carried out the disclosure, who was present, reasons, details, and important points when disclosing

Items are the same as state in Q2, Q3, Q4 and Q5 above.

Q12 Whether or not the genetic condition was discussed with the siblings after disclosure

Items:

1. Always (if possible, please explain reasons)
2. Sometimes (if possible, please explain reasons)
3. Never (if possible, please explain reasons)

Q13 Will you disclose to the siblings later, if you haven't yet?

Items:

1. Yes 2. No

Q14 When will you disclose to the siblings?

Q15 Your plan to disclose to the siblings: age at disclosure, who will primarily carry out the disclosure, who will be present, reasons, details, and important points when disclosing

(Items are the same as state in Q2, Q3, Q4 and Q5 above)

Questionnaire II. For respondents who had not disclosed information to their children

---

Q1 Reasons for avoiding disclosure

Items:

1. Had never been asked about their symptoms
2. Had never been asked why he/her had to visit the hospital
3. Didn't think he/she was mature enough to understand
4. Thought it would be a psychological burden to the child to know the status
5. Didn't think he/she seemed to be concerned about physical symptoms
6. Because friends of his/her didn't seem to be bothered by his/her symptoms
7. Because there has not been any one of school enrollment, employment, or marriage, etc., yet.
8. Didn't want children to know
9. Wanted to postpone telling it until as late as possible
10. There has not been a need to unveil
11. Others (If possible, please explain in detail)

Q2 Whether or not you will disclose information in the future

Items:

1. Plan to disclose the diagnosis in the future
2. Do not disclose
3. Not sure

Q3 If you plan on disclosing in the future: the age at disclosure, who will primarily carry out the disclosure, who will be present, reasons, details, and important points when disclosing.

(Items are the same as stated in Q2, Q3, Q4 and Q5 on questionnaire I)

Q4 Number of siblings of the child with a genetic condition

Q5 Whether parents disclosed information about genetic conditions to siblings or not

Items:

1. Yes 2. No

Q6 The age of siblings at disclosure, who primarily carried out the disclosure, who was present, reasons, details, and important points when disclosing, OR your plan to disclose information to the siblings

(If yes above the Q5 question, items are the same as stated in Q2, Q3, Q4 and Q5 on questionnaire I.)

---

[1] Respondents were asked to indicate the degree of their feelings by choosing any number on a scale
